# Systematic evaluation of TaqMan real-time polymerase chain reaction assays targeting the *dsb* and *gltA* loci of *Ehrlichia canis* in recombinant plasmids and naturally infected dogs

**DOI:** 10.14202/vetworld.2022.701-706

**Published:** 2022-03-25

**Authors:** Peeravit Sumpavong, Wanat Sricharern, Natnaree Inthong, Gunn Kaewmongkol, Sarawan Kaewmongkol

**Affiliations:** 1 Department of Veterinary Technology, Faculty of Veterinary Technology, Kasetsart University, Bangkok, Thailand; 2 Department of Companion Animals Clincial Sciences, Faculty of Veterinary Medicine, Kasetsart University, Bangkok, Thailand

**Keywords:** detection, diverse local genotypes, *Ehrlichia canis*, TaqMan real-time polymerase chain reaction

## Abstract

**Background and Aim:**

Because of the diversity of local genotypes of *Ehrlichia canis*, genes targeted by TaqMan real-time polymerase chain reaction (RT-PCR) assays should be systematically evaluated. This study evaluated the amplification efficiency, linearity, precision, and sensitivity of two TaqMan RT-PCR assays targeting the *dsb* and *gltA* loci of *E. canis* in recombinant plasmids and naturally infected dogs.

**Materials and Methods:**

Thirty blood samples were collected from dogs showing clinical signs of canine monocytic ehrlichiosis at the Kasetsart University Veterinary Teaching Hospital, Bangkok, Thailand. The *dsb* and *gltA* genes were amplified by conventional PCRs (cPCRs) on the blood samples and were then sequenced. Meanwhile, RT-PCR was used to detect *dsb* and *gltA* genes in 10-fold dilutions of the recombinant plasmids.

**Results:**

Both *dsb* and *gltA* were amplified with a high degree of linearity (*R^2^*≥0.975 and 0.993, respectively) in all dilutions, although the mean percentage of relative standard deviation of *glt*A was lower, but the difference was non-significant. The detection limits of RT-PCR and cPCR were 10^−7^ and 10^−6^, respectively, for both loci. RT-PCR targeting *dsb* (22/30; 73.3%) and *gltA* (15/30; 50%) yielded a number of positive results that did not differ significantly (p=0.06). The RT-PCR positive results of the *dsb* gene (22/30) differed significantly from that of cPCR (11/30) (p=0.004). In contrast, the RT-PCR positive results of the *gltA* gene (15/30) did not differ significantly from that of cPCR (12/30) (p=0.43). The mean Ct value (30.2) based on *dsb* RT-PCR of 22 positive cases was higher than that of *gltA* RT-PCR (Ct=27.4) on 15 positive cases. The Ct values from *dsb* RT-PCR were >30 in all seven discordant samples that were not detected by the *gltA* RT-PCR.

**Conclusion:**

RT-PCR targeting the *dsb* gene was more sensitive for detecting *E. canis * in naturally infected dogs. This study suggested that TaqMan RT-PCR of the *dsb* gene should be selected for *E. canis* research in this region.

## Introduction

*Ehrlichia canis* is a tick-borne bacterium that targets monocytes and causes canine monocytic ehrlichiosis (CME) [1]. Domestic dogs are the major host of *E. canis*, although this bacterium can infect other canids and felids [2,3]. CME has spread globally and causes death in severe cases [4]. The disease has also been reported in humans [5]. CME is a multisystemic disease with acute, chronic, and subclinical forms. Acute CME is characterized by high fever, depression, lethargy, anorexia, lymph node enlargement, and splenomegaly [6]. Diagnosis of CME is based on hematological, biochemical, and serological findings, and the pathogen is identified by microscopic examination [7]. At present, the immunofluorescence antibody assay is considered the “gold standard” [8] for serological detection of *E. canis *, and many researchers have used this method to detect immunoglobulin G associated with *E. canis* infection. However, this method is limited by known cross-reactive antibodies [9].

Conventional polymerase chain reaction (cPCR) and nested polymerase chain reaction (PCR) are rapid, sensitive, and specific methods for the precise diagnosis of ehrlichiosis [10]. However, both methods have their disadvantages. For example, both methods generate PCR product contamination during the agarose gel electrophoresis stage. Real-time PCR (RT-PCR) detection can save time and obviate the need for agarose gel electrophoresis. RT-PCR and DNA sequencing are the most reliable detection tools for *E. canis* in blood and tissue samples [11]. Most importantly, the RT-PCR assay provides quantitative estimates of the load of the organism in infected animals [12]. Probe-based RT-PCR assays are also highly specific and generate less contamination than RT-PCR, which relies on intercalating dyes [13]. There are two major reference genes that have provided highly sensitive and specific results using probe-based RT-PCR: The *Ehrlichia* disulfide bond formation protein gene (*dsb*) [14] and the citrate synthase gene (*gltA*) [15].

In Thailand, DNA sequence analyses of the *gp36* gene have confirmed the genetic diversity of bacterial strains and elucidated geographical patterns of the local genotypes [16-18]. The target genes that have been used for RT-PCR are extremely diverse, and there has been no systematic evaluation of genes that are suitable for the detection of local genotypes of *E. canis* by RT-PCR in Thailand and Southeast Asia.

This study aimed to evaluate two TaqMan RT-PCR assays targeting the *dsb* and *gltA* loci of *E. canis* by estimating the amplification efficiency, linearity, precision, and sensitivity of each assay.

## Materials and Methods

### Ethical approval

This study was approved by the Animal Ethics Committee of the Faculty of Veterinary Medicine, Kasetsart University, Bangkok, Thailand.

### Study period and location

The study was carried out from November 2020 to May 2021. Blood samples were collected from 30 dogs in Kasetsart University Veterinary Teaching Hospital, Bangkok, Thailand.

### Blood sample collection and DNA extraction

Two milliliters of whole blood were collected from each dog displaying possible clinical signs of ehrlichiosis (pale mucous membrane, petechia, ecchymosis, fever, and anorexia) and harboring ticks. In addition, there were abnormalities of blood parameters including anemia, thrombocytopenia, or pancytopenia. The whole blood samples were kept in EDTA (4.55 mmol per liter of blood) and stored at 4°C until extraction of genomic DNA using an E.Z.N.A. Tissue DNA kit (Omega Bio-Tek, Norcross, USA) following the manufacturer’s protocol without modification. The DNA extracts were stored at −20°C.

### cPCR amplification and DNA sequencing

The same oligonucleotide primers targeting the *dsb* and *gltA* genes were used for cPCR, RT-PCR, and DNA sequencing (Table-1) [10,15]. For cPCR, the reaction mixture contained 1 pmol of the forward and reverse primers, 0.2 μL dNTP, 2.5 mM MgCl_2_, 0.2 U *Taq* DNA polymerase with buffer (Invitrogen, Carlsbad, CA, USA), and 2 μL of extracted DNA. The cycling conditions for both assays consisted of initial denaturation at 95°C for 5 min, followed by 35 cycles at 95°C for 1 min, 55°C for 30 s, 72°C or 45 s, and ending with a final extension at 72°C for 10 min. The PCR products were verified by 1.5% (w/v) agarose gel electrophoresis. PCR products of the expected sizes were purified using an UltraClean^®^15 DNA purification kit (MO BIO Laboratories, Inc., Carlsbad, CA, USA). The purified amplicons were sequenced by First BASE Laboratories (Sdn Bhd, Selangor, Malaysia).

**Table-1:** Primers used for the amplification and sequencing of the *dsb* and *gltA* genes.

Target	Primer	Sequence	Product size (bp)	Reference
*dsb*	DSB-321	5’- TTGCAAAATGATGTCTGAAGATATGAAACA-3’	350	[10]
	DSB-671	5’- GCTGCTCCACCAATAAATGTATCYCCTA-3’		
*gltA*	gltA-Forward	5’- TAGCAACTTTATGGGGGCCA-3’	146	[15]
	gltA-Reverse	5’- TGACCAAAACCCATTAGCCTC-3’		

### Cloning of *dsb* and *gltA*

Amplicons of the *dsb* and *gltA* genes (350 and 150 bp, respectively) were cloned into the pGEM-T Easy vector system I (Promega, Madison, WI, USA), according to the manufacturer’s instructions. The ligation products were transformed into *Escherichia coli* DH5a competent cells. Transformants were grown and their plasmid DNA was extracted using a GeneJET Plasmid Miniprep Kit (Thermo Fisher Science, Waltham, MA, USA). The DNA concentrations of the pGEMT-*dsb* and pGEMT-*gltA* plasmids were measured using a Nanodrop 2000c Spectrophotometer (Thermo Fisher Science, Waltham, MA, USA). Plasmid concentration values of pGEMT-*dsb* and pGEMT*-gltA* were used to calculate the plasmid copies/mL as previously described [19], and their initial concentrations were set to 400 and 398 ng/μl, respectively. The pGEMT-*dsb* and pGEMT-*gltA* plasmids were serially diluted from 1×10^−3^ to 1×10^−8^ copies/μL, and the dilution series were used to generate two standard curves. Each curve is the result of triplicate serial dilutions (i.e., n=3/plasmid).

### RT-PCR

The RT-PCR reaction mixture (20 μL) contained 10 μL of ×5 HOT FIREPol Probe qPCR Mix Plus (Solis BioDyne, Tartu, Estonia), 1 μM of each primer, and the TaqMan probes listed in Table-2 [10,15]. The RT-PCR was carried out on an RT-PCR CFX96 machine (Bio-Rad Laboratories, Hercules, CA, USA). The PCR cycling conditions consisted of 95°C for 15 min, followed by 45 cycles of 95°C for 15 s and 60°C for 1 min.

**Table-2:** TaqMan probe used for real-time polymerase chain reaction.

TaqMan probe	Sequence	Reference
*dsb* TaqMan probe	FAM-5’- AGCTAGTGCTGCTTGGGCAACTTTGAGTGAA-BHQ1-3’	[10]
*gltA* TaqMan probe	FAM-5’- AGTAACGTAAAGCAGTTTATTCAA-BHQ1-3’	[15]

### Amplification efficiency, analytical sensitivity, and specificity

The amplification efficiency and analytical sensitivity of the TaqMan RT-PCR and cPCR assays were tested on a 10-fold dilution series of a recombinant plasmid. Three replicates were evaluated per dilution of copy number and test. The sensitivity of each amplification was scored by the lowest dilution of each plasmid, and the amplification efficiency for the TaqMan assay was calculated from logarithmic regression analysis of the respective data [20]. Real-time PCR efficiencies of *dsb* and *gltA* were calculated from the given slopes in CFX Maestro Software for Bio-Rad CFX RT-PCR 96 units. The RT-PCR efficiency (E) of one cycle in the exponential phase was calculated according to the equation;

E=10^(-1/slope)^.

The specificities of the two assays were assessed by performing each assay on whole blood specimens collected from 30 dogs that were suspected of *E. canis* infection. All positive results were confirmed by sequencing the resulting amplicons.

### Statistical analysis

Statistical analyses were performed using the SPSS 23.0 for Windows software package (IBM SPSS Statistics, Chicago, IL, USA). The linear coefficient of determination (*R^2^*) and the percentage of relative standard deviation (RSD; % = (standard deviation/mean Ct value) × 100) were calculated from 10-fold dilution series of recombinant plasmids in Ct values of both genes, triplicate measurements, and over three separate runs. Chi-squared analysis was used to compare the cPCR- and RT-PCR-positive samples based on the amplification of the *dsb* and *gltA* genes. Differences were considered significant at p*<*0.05.

## Results

### Amplification efficiency, sensitivity, and specificity

**Figure-1:**
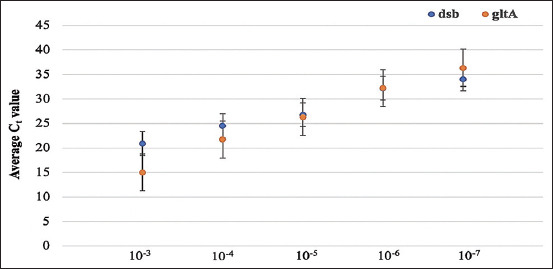
Regression analysis for TaqMan amplification from 10-fold serial dilution of pGEMT-*dsb* and pGEMT-*gltA*. The mean cycle threshold (Ct) value is plotted against the known dilutions and the error bars show the standard deviation around this value.

The standard curve generated by RT-PCR targeting pGEMT-*dsb* was represented by the equation: *Y*=−3.408*X*+48.118, where *Y* is the cycle threshold (Ct) and *X* is the log copy number of the DNA target. Meanwhile, the standard curve generated by RT-PCR targeting pGEMT-*gltA* was represented by the equation: *Y*=−5.317*X*+52.924. The correlation coefficients (*R^2^*) for the equations are 0.975 and 0.993, respectively (Figure-1).

The amplification efficiencies using the TaqMan primer sets were assessed on 10-fold serial dilutions of recombinant plasmids. The calculated efficiencies of the TaqMan assays for the *dsb* and *gltA* genes are well within the tolerance limits (E=96.5% and 54.19%, respectively). Furthermore, average Ct values from three independent runs to 10-fold serial dilutions of plasmid DNA of the *dsb* and *gltA* genes generated a linear equation with an R2 equal to (1) (0.975 and 0.993, respectively) for both assays (Figure-1). The sizes of the RT-PCR amplicons of *dsb* and *gltA* were 350 and 150 bp, respectively, based on gel electrophoresis (data not shown). The TaqMan assays were more sensitive than cPCR, as the former were able to amplify target templates diluted to as low as 1×10^−7^, while the latter amplified templates diluted to as low as 1×10^−6^ (Table-3). The identities of the amplicons generated by the two PCR assays were successfully confirmed by DNA sequencing.

**Table-3:** Sensitivity of conventional and TaqMan real-time PCR.

Plasmid 10-fold dilution	cPCR		TaqMan real-time PCR	
	
	*Dsb*	*gltA*	*dsb*	*gltA*
10^-1^	+	+	+	+
10^-2^	+	+	+	+
10^-3^	+	+	+	+
10^-4^	+	+	+	+
10^-5^	+	+	+	+
10^-6^	+	+	+	+
10^-7^	-	-	+	+
10^-8^	-	-	-	-

PCR=Polymerase chain reaction, cPCR=Conventional polymerase chain reaction

**Figure-2:**
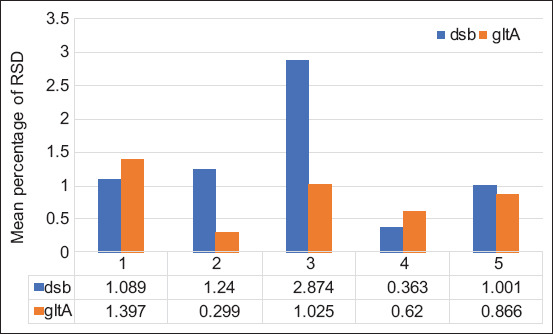
Comparison of the precision of real-time PCR on cloned *E. canis* targets (gene copy numbers ranged from 10^-3^ to 10^-7^) for *dsb* and *gltA* gene plasmid construct as measured using the percentage of relative standard deviation (RSD). The data show the average results from three separate runs.

The precision of each assay was evaluated by analyzing the mean % RSD based on triplicate measurements performed on three separate runs. Overall, the % RSD values of the pGEMT-*gltA* assay was lower than that of pGEMT-*dsb* (Figure-2). However, the difference was not significant. The precision of the pGEMT-*dsb* assay appeared unaffected by the DNA concentration.

### Comparison of conventional and TaqMan RT-PCR in 30 blood samples

The presence of *E. canis* in 30 blood samples based on the amplification of two target genes by conventional PCR (cPCR) and RT-PCR is shown in Table-4. The results of RT-PCR targeting *dsb* and *gltA* were inconsistent for seven blood samples. Fifteen samples tested positive for both *gltA* RT-PCR and *dsb* RT-PCR. Seven samples tested positive for *dsb* RT-PCR but yielded negative results for *gltA* RT-PCR. The mean Ct values for *dsb* (22 positives) and *gltA* (15 positives) TaqMan PCR results were 30.2 and 27.4, respectively. For the seven samples for which the results of the two assays were inconsistent, the Ct values for *dsb* PCR were >30.

**Table-4:** Number of positive samples detected using cPCR and TaqMan real-time PCR.

Type	Number	cPCR		TaqMan real-time PCR	
		*Dsb*(%)	*gltA*(%)	*dsb*(%)	*gltA*(%)
Whole blood	30	11 (36.67)	12 (40)	22 (73.33)	15 (50)
Mean Ct value				30.2	27.4

PCR=Polymerase chain reaction, Ct=Cycle threshold, cPCR=Conventional polymerase chain reaction

The rates of the detection of *E. canis* in the 30 blood samples did not differ significantly between *dsb* (22/30; 73.3%) and *gltA* (15/15; 50%) RT-PCRs (p=0.06307), which agree with the *dsb* (11/30; 36.67%) and *gltA* (12/30; 40%) cPCR results (p=0.7906). The rate of detection of *E. canis* through *dsb* RT-PCR (22/30; 73.3%) was significantly higher than that for *dsb* cPCR (11/30; 36.67%) (p=0.004), as shown in Table-5. In contrast, the RT-PCR results for the *gltA* gene (15/30; 50%) did not differ significantly from that of the *gltA* cPCR (12/30; 40%) (p=0.43), as shown in Table-6.

**Table-5:** Detection results for *E. canis* detection using *dsb* gene compared between TaqMan real-time PCR and cPCR.

	cPCR	TaqMan real-time PCRs	Marginal row totals
*dsb* positive	11 (1.83)	22 (1.83)	33
*dsb* negative	19 (2.24)	8 (2.24)	27

Chi-square statistic is 8.1481 and p-value is 0.004311 (p<0.05). *E. canis=Ehrlichia canis*, PCR=Polymerase chain reaction, cPCR=Conventional polymerase chain reaction

**Table-6:** Detection results for *E. canis* detection using *gltA* gene compared between TaqMan real-time PCR and cPCR.

	cPCR	TaqMan real-time PCRs	Marginal row totals
*gltA* positive	12 (0.17)	15 (0.17)	27
*gltA* negative	18 (0.14)	15 (0.14)	33

Chi-square statistic is 0.6061 and p-value is 0.436275 (p<0.05). *E. canis=Ehrlichia canis,* PCR=Polymerase chain reaction, cPCR=Conventional polymerase chain reaction

## Discussion

This study aimed to evaluate two TaqMan RT-PCR assays targeting the *dsb* and *gltA* loci of *E. canis* by estimating the amplification efficiency, linearity, precision, and sensitivity of each RT-PCR assay. Based on the amplification results of plasmid standards, the RT-PCR assays for both genes had similar sensitivities. Using the probes and primer sets on local target genotypes of *E. canis* in Thailand, we confirmed the specificity of each target gene by DNA sequencing; we found no false-positive results throughout the evaluations. All positive samples were confirmed as *E. canis* by DNA sequencing.

Implementing the TaqMan RT-PCR assay will decrease the time needed to make clinical decisions and reduce the cost of investigations because the need to perform gene sequencing to verify the PCR products is obviated. Additional advantages of the RT-PCR assays are as follows: They are quicker to set up and require fewer pipetting steps, thus reducing the possible sources of error and contamination.

The generation of standard curves and the determination of the efficiencies of the primers and probes are critical to obtaining accurate results when using RT-PCR assays. In the present study, the *dsb* and *gltA* genes were amplified with high degrees of linearity from plasmid standards (*R^2^*≥0.975 and 0.993, respectively). However, *gltA* was not a better choice for the detection of *E. canis*, because its amplification efficiency was 54.19% that was significantly lower than that for the *dsb* gene (96.5%). The acceptable efficiency should be within 90 to 105% [21]. In addition, the *dsb* RT-PCR assay was able to detect more pathogens from the same set of samples than the *gltA* RT-PCR assay (Table-4). As expected, RT-PCR and cPCR targeting *dsb* yielded significantly different results (Table-5). However, the results of RT-PCR and cPCR targeting *gltA* did not differ significantly (Table-6).

Using plasmid standards, we demonstrated that the specificities and sensitivities of the *gltA* and *dsb* genes for *E. canis* detection were similar. However, when applied to natural infections, the *dsb* gene is likely to be more sensitive than the *gltA* gene.

RT-PCR amplifications were performed using total DNA from plasmid standards and blood samples. Using linear regression, we derived the following equation [22] to determine the quantity of the target DNA templates in each unknown sample:

N_n_=_,_ where n=C_t_


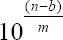



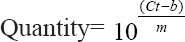


In general, Ct values increase as the quantity of DNA template decreases (relative bacterial load). Amplification of the *gltA* gene had a lower mean Ct value, which may be due to the low efficiency of amplification of this gene. Therefore, the TaqMan RT-PCR of the *gltA* could successfully detect *E. canis* DNA only in blood samples with a high relative bacterial load. The *dsb* gene was a more effective target than the *gltA* gene for detecting low levels of *E. canis* DNA . Therefore, the PCRs should be performed under the same reaction conditions to reliably compare Ct values. Ct values from PCR reactions performed under different conditions or with different reagents should not be directly compared.

The use of an oligonucleotide probe in TaqMan RT-PCR increases the level of sequence-based specificity of pathogen detection. However, target gene polymorphisms may greatly affect the sensitivity of the technique [23]. False-negative results are common, due to the occurrence of mismatches in the probe-binding region [24,25]. Our systematic evaluation of whole blood samples collected from dogs indicates that the assay targeting the *dsb* gene is the more sensitive method. The RT-PCR assay targeting the *gltA* gene in naturally infected dogs yielded unexpected results as no significant difference of a positive number between RT-PCR and cPCR targeting the *gltA* gene. A study of uncultured *Ehrlichia* bacteria in *Haemaphysalis* ticks in Japan revealed polymor­phisms of the *gltA* loci [26]. DNA sequence alignments of the *gltA* and citrate synthase gene primer sets of *Anaplasma platys* (GenBank accession number EU516387) and *E. canis* found 75% similarity at the binding sites of both forward and reverse primers targeting the *gltA* gene of both species. These two bacteria frequently coinfect dogs [27,28]. Therefore, the *gltA* primers used in our study can likely amplify the genes of both *E. canis* and *A. platys*. The sequences of the probe-binding regions of these two species also differed by 46%, indicating the possibility of *gltA* RT-PCR generating false-negative results.

A sufficiently large genetic data set of members of the family Anaplasmataceae is required to enhance the performance of PCR and qPCR tools. However, GenBank contains only a few sequences of housekeeping genes of members of the family Anaplasmataceae in Southeast Asia. Analyses of whole-genome sequences, as well as sequences of the 16S ribosomal RNA and other housekeeping genes, indicate that *Ehrlichia, Anaplasma, Neorickettsia, Neoehrlichia, Wolbachia, Orientia*, and *Rickettsia* have evolved from a common ancestor [29,30]. Therefore, the target genes used in our study may contain regions that are conserved among the members of this family, which may cause non-specific amplification that lowers the sensitivity of the *gltA* RT-PCR in naturally infected dogs.

## Conclusion

Our systematic evaluation of two RT-PCR assays applied to naturally infected whole blood sample of unknown dogs indicate that, based on sensitivity and specificity, RT-PCR targeting the *dsb* gene is more suitable for the identification of *E. canis* in this geographical area. The accurate identification of *E. canis* by RT-PCR can improve the quality of research and detection efficacy of CME in tropical regions. We recommend the standardization of TaqMan RT-PCR protocols to detect local genotypes of *E. canis* in tropical regions. We have demonstrated the advantage of this approach as applied to real, naturally infected dogs. Limitations of the study includes a small number of the infected dogs and an insufficient genetic data set of members of bacteria in the family Anaplasmataceae in this region. The reliable RT-PCR of *E. canis* can be applied for further studies of novel treatments and alternative clinical specimens for molecular detection.

## Authors’ Contributions

SK and WN: Designed the study and revised and finalized the manuscript for submission. PS and GK: Designed and conducted the study, interpreted the results, and drafted the manuscript. NI: Performed the study and reviewed the manuscript. All authors read and approved the final manuscript.
